# Indocyanine green fluorescence improves safety in laparoscopic cholecystectomy using the Fundus First technique: a retrospective study

**DOI:** 10.3389/fsurg.2025.1516709

**Published:** 2025-01-23

**Authors:** Susanna Haverinen, Evelina Pajus, Gabriel Sandblom, Yücel Cengiz

**Affiliations:** ^1^ Department of Clinical Science and Education, Södersjukhuset, Karolinska Institute, Stockholm, Sweden; ^2^ Department of Surgery, Sundsvall General Hospital, Sundsvall, Sweden; ^3^ Department of Diagnostics and Intervention, Surgery, Umeå University, Umeå, Sweden; ^4^ Department of Surgery, Södersjukhuset, Stockholm, Sweden

**Keywords:** indocyanine green, fluorescent cholangiography, laparoscopic cholecystectomy, Fundus First, cholangiography

## Abstract

**Introduction:**

As one of the most commonly performed surgeries in the world, safety during laparoscopic cholecystectomy (LC) is of utmost importance. Indocyanine green (ICG) has been used for different medical purposes including assessment of liver function since the 1950s. Its use during LC was first described in 2009 by Ishizawa. Since ICG is excreted in the bile, its fluorescent properties can be used to illuminate the bile ducts, and may reduce the risk for bile duct injury and other complications. Previous studies have compared ICG with conventional visualization showing shorter operation time and lower conversion rates during LC performed with traditional operation techniques. Results from LC performed with the Fundus First method (FF-LC) and ICG fluorescence has not been previously reported. The aim of this retrospective study was to compare LC with and without the aid of ICG fluorescence at a Swedish hospital routinely performing FF-LC.

**Methods:**

Data from all patients operated with LC at Sundsvall General Hospital before and after the implementation of routine ICG between 2016 and 2023 were analyzed.

**Results:**

The study included 2,009 patients; 1,455 operated with ICG (ICG-group) and 549 without (comparison group). FF-LC was used in 94.9% of all operations. The groups were comparable regarding gender, BMI, age, presence of acute cholecystitis and proportion urgent/elective surgery. ICG was found to be safe, with similar 30-day complication rates between study groups. A lower conversion rate was seen in the ICG-group (1.2% vs. 3.3%, *p* = 0.001) and there was a non-significant reduction in readmissions (*p* = 0.054). In univariate analysis, ICG was associated with prolonged operation time, but this was not supported in multivariate analysis. Time to cholangiography was prolonged in the ICG-group in both univariate and multivariate analyses.

**Discussion:**

ICG fluorescence is an adjunct that could improve the operative safety. Implementation of routine ICG fluorescence at this Swedish hospital was found to be safe and efficient, suggesting improvement in safety during FF-LC. Further studies are needed to see if ICG increases safety in LC.

## Introduction

Surgery for gallstone disease is a common procedure in Europe (1, 2). In Sweden, approximately 14,000 cholecystectomies are performed annually, with over 90% carried out laparoscopically (3). Laparoscopic cholecystectomy (LC) has become the gold standard providing faster recovery, shorter length-of-stay and lower morbidity than open cholecystectomy (1, 4, 5).

The risk for bile duct injury (BDI) during LC is a great concern (6). BDI is regarded as one of the major quality measures of safe LC, with reported rates between 0.03 to 2.6% (7–11). This serious complication has a high morbidity often requiring subsequent surgical treatment and, in severe cases, liver transplantation, making it of utmost clinical importance (8, 12–14).

Misinterpretation of the anatomy of the biliary tract is a common cause of BDI in LC, and various strategies to improve tract identification with the aim of reducing BDI have been tried (7, 15). The “Critical View of Safety” (CVS) is generally applied to ensure correct anatomical identification before dividing the cystic duct (16). A laparoscopic cholecystectomy performed with the Fundus First method (FF-LC), with dissection beginning at the gallbladder fundus, has been suggested to offer additional safety in difficult situations (17). In a meta-analysis the FF-LC reduced the risk for conversion to open surgery, operation time, and risk for BDI in difficult cholecystectomies (18). A randomized controlled trial (RCT) found it cost-effective when compared to traditional LC with electrocautery (19), and these results were confirmed when FF-LC was introduced as the standard procedure (20).

Intraoperative cholangiography (IOC) is used to identify anatomical variations, bile duct stones, and BDI (21). However, IOC also carries a risk since cannulation of the cystic duct can injure the bile duct. The radiation involved, prolonged operation time, and increased expense are other disadvantages that make its routine use debatable (22). A systematic review did not find routine IOC to be beneficial in low-risk patients, and it is not recommended by the European Association for the Study of the Liver, EASL (1, 23). In Sweden, most centers preform IOC as a routine and IOC was performed in 89.2% of elective and 90.7% of urgent LCs in 2022 (3). This routine is supported by previous Swedish studies showing benefits of routine IOC (10, 24, 25). Earlier detection of BDI and reduced consequences of the BDI have been shown, in addition to an improved survival by the intension to use IOC (10, 24). As a result, analyses have shown IOC to be cost-effective (25).

Indocyanine green (ICG) fluorescence for the visualization of the biliary tract was first described by Ishizawa in 2009 (26). ICG is a dye almost exclusively excreted in the bile and is fluorescent when illuminated with near-infrared light (27). The ICG fluorescence penetrates a few millimeters of the tissue (27). The substance is injected prior to LC and near-infrared light illuminating the biliary tract is applied during the procedure. Studies have suggested that ICG may be as effective as IOC for visualizing critical anatomical landmarks during LC (28–31). In an RCT on laparoscopic cholecystectomy using the conventional dissection technique, identification was significantly superior with ICG for most structures though the effect became less with increasing BMI (31).

Operation times, with and without ICG, have not differed in some studies (28, 32, 33), though others have found significantly reduced operation times (7, 34–37), even in pediatric patients (38). A multicenter RCT found shorter time to CVS and shorter operation time when using ICG fluorescence (39) whereas another found longer operation times (40).

Better visualization of the biliary tract should result in less need for conversion to open surgery. This was demonstrated in a retrospective study of 1,389 patients, finding a conversion rate of 1.5% with ICG vs. 8.5% without (*p* < 0.0001) (7). The same was seen in a study on percutaneous transhepatic gallbladder drainage (37).

Previous studies evaluating ICG have involved traditional LC with dissection starting from Calot's triangle, or in settings where the operation approach was not described or heterogeneous. ICG fluorescence combined with FF-LC has been routinely used at Sundsvall General Hospital since 2016. Comparison of FF-LC with and without ICG has, to our knowledge, not been reported. The aim of this retrospective study was to compare outcomes of FF-LC following the implementation of routine ICG fluorescence at a Swedish general hospital.

## Methods

### Data collection

A retrospective study on all adult patients treated with urgent or elective LC at Sundsvall General Hospital between 1st January 2015 and 31st August 2023. This period began the year before the introduction of ICG and onwards. Patients below 18 years of age were excluded.

Patients were identified through the Swedish Gallstone Surgery and Endoscopic Retrograde Cholangiopancreatography Register, GallRiks. This register is continually validated and has a coverage of over 90% of gallstone surgeries in Sweden (38, 39). Additional information was collected from patient records.

The study was approved by the Ethics Review Board (reference number 2023-02657-01).

### Surgical technique

Since 2000, laparoscopic cholecystectomies have been performed at Sundsvall General Hospital using the Fundus First method (FF). Under general anesthesia, open-entry access (Hasson) below the umbilicus is achieved followed by ports via four standard locations. The laparoscopic systems used was ICG compatible staples by KARL STORZ™ (Tuttlingen, Germany). Intra-abdominal pressure was kept at 12–15 mmHg. A ultrasonic dissector by Ethicon™ (Norderstedt, Germany) was used for dissection, set at level 3/5. After initial exploration of the field and removal of adhesions, the peritoneum surrounding the infundibulum was opened and the gallbladder dissected from the liver from the fundus towards the peritoneal opening. Intraoperation cholangiography was always attempted. The cystic duct was divided between metallic clips and gallbladder extracted via the umbilical port in a specimen bag. The cystic artery was usually divided by ultrasonic shears or between metallic clips.

We used Verdye™ indocyanine green at a concentration of 5 mg/ml and a dose of 0.1 mg/kg body weight. This is administered intravenously 60–120 min prior to surgery in elective cases, and in urgent cases when the decision to operate is made.

### Registered parameters

Data acquired from GallRiks were patient characteristics (age, gender, BMI, comorbidity), operation data (date of surgery, inpatient/day-case, acute/elective, surgical method, findings at IOC, perioperative ERCP) and postoperative data (complications within 30 days and readmission). Data missing in GallRiks and time for ICG administration were retrieved from the medical records. Time to cholangiography was calculated as time from start of surgery to the time shown on the cholangiography images.

### Statistical analysis

Descriptive statistics were performed using either Chi Square test or Fisher's Exact test for nominal data. Student's *T* test was used for scale variables to compare means of binary variables, and ANOVA was used if more than two comparators existed. Mann–Whitney *U* test was used for non-normally distributed data. Levene's test was used to see if variance was equal between groups when using the Student's *T* test.

Multiple logistic regression analysis was performed to identify predictors for conversion, and odds ratios (OR) for conversion with 95% confidence intervals were calculated. Besides the use of ICG, age and urgent/elective surgery were selected as independent variables in univariable analysis.

Multiple linear regression analysis was performed to examine if ICG use was independently correlated with increase/decrease in operation time and time to IOC. All multiple regression analyses were considered exploratory.

Statistical analyses were preformed using SPSS Statistics version 29.0.2.0 (IBM Corp., Armonk, NY, United States).

## Results

### Patient characteristics

A total of 2,009 patients were included in the study of which ICG fluorescence was used in 1,455 cases. Baseline data are presented in Table 1. In 1,032 cases, the time of administration of ICG was documented (median 137 min prior to surgery). Data regarding use of ICG was missing for five patients (0.2%). The groups differed with lower mean age, higher proportion of smokers, and more patients with cardiovascular disease in the non-ICG group.

**Table 1:** Baseline characteristics of patients operated with and without ICG with *p*-values accordingly. Numbers in parentheses are corresponding percentages if not otherwise stated.

	With ICG	Without ICG	Total	Missing	*p*
	1,455 (72.4)	549 (27.3)	2,009 (100)	5 (0.2)	
Gender				5 (0.2)	0.78
Male	544 (72.2)	209 (27.8)	753 (37.6)	1 (0.05)	
Female	911 (72.8)	340 (27.2)	1,251 (62.4)	4 (0.2)	
Mean age, years (SD)	52.6 (16.5)	49 (16.2)	2,009 (100)	5 (0.2)	<0.001
Diabetes	110 (7.6)	30 (5.5)	140 (7)	12 (0.6)	0.1
Cardiovascular disease	231 (16)	114 (20.8)	345 (17.3)	12 (0.6)	0.01
COPD/Asthma	110 (7.6)	53 (9.7)	163 (8.2)	14 (0.7)	0.1
Increased bleeding risk^a^	84 (5.8)	25 (4.6)	109 (5.5)	12 (0.6)	0.3
Immunosuppressive agents	25 (1.7)	8 (1.5)	33 (1.7)	12 (0.6)	0.7
ASA-class				12 (0.6)	0.002
1	456 (31.5)	202 (36.8)	658 (32.9)		
2	778 (53.7)	295 (53.7)	1,073 (53.7)		
3	205 (14.2)	50 (9.1)	255 (12.8)		
4	9 (0.6)	1 (0.2)	10 (0.5)		
5	0 (0)	1 (0.2)	1 (0.1)		
Smoking	56 (4.1)	43 (9.3)	99 (5.4)	178 (8.9)	<0.001
BMI, mean (standard deviation)	29.3 (5.6)	29.0 (5.2)	1,584 (78.8)	425 (21.2)	0.33
Acute cholecystitis	397 (27.3)	145 (26.4)	542 (27)	5 (0.2)	0.7
Urgent surgery	587 (40.3)	229 (41.7)	816 (40.7)	5 (0.2)	0.58
Form of care				5 (0.2)	0.47
Inpatient	626 (43)	246 (44.8)	872 (43.5)		
Day-case	829 (57)	303 (55.2)	1,132 (56.5)		
Type of surgery				5 (0.2)	0.004
Laparoscopic cholecystectomy	1,427 (98.1)	525 (95.6)	1,952 (97.4)		
Laparoscopic converted to open	17 (1.2)	18 (3.3)	35 (1.7)		
Subtotal LC	11 (0.8)	5 (0.9)	16 (0.8)		
Other	0	1 (0.2)	1 (0.0)		
Type of dissection				18	0.016
Fundus first	1,369 (94.7)	521 (95.6)	1,890 (94.1)		
Traditional LC	68 (4.7)	15 (2.8)	83 (4.13)		
No dissection before conversion	9 (0.6)	9 (1.7)	18 (0.9)		
Year of surgery, *n* (percent of surgeries for the corresponding year)				5 (0.2%)	<0.001
2015	0 (0)	157 (100)	157		
2016	26 (14.3)	156 (85.7)	182		
2017	102 (44.2)	129 (55.8)	231		
2018	194 (77)	58 (23.0)	252		
2019	220 (91.3)	21 (8.7)	241		
2020	286 (97.3)	8 (2.7)	294		
2021	246 (95.3)	12 (4.7)	258		
2022	234 (98.3)	4 (1.7)	238		
2023	147 (97.4)	4 (2.6)	151		

ASA, physical status according to American Society of Anesthesiologists; BMI, Body Mass Index; COPD, Chronic Obstructive Pulmonary Disease; ICG, Indocyanine green; LC, Laparoscopic Cholecystectomy; SD, Standard Deviation.

^a^ Increased bleeding risk defined as patients with routine use of an anticoagulant and-/or thrombocyte inhibitor or with a prolonged coagulation time due to hematological disease.

### Operation data and complications

The FF method was used in 95.6% of operations without ICG and in 94.7% of those with ICG fluorescence. A higher proportion of operations without ICG were converted before any dissection was performed compared to operations with ICG (1.7% and 0.6% respectively, *p* = 0.016) (see Table 2).

**Table 2:** Operation data, complications, and readmissions for patients operated with and without ICG, respectively. Numbers in parentheses are corresponding percentages if not otherwise stated.

	With ICG	Without ICG	Missing	*P*
Total	1,455 (72.6)	549 (27.4)	5	
Mean operation time, min (SD)	72.6 (36.3)	65.8 (37.5)	0	<0.001
Mean operation time^a^, min (SD)	64.1 (24) (*n* = 1,311)	57.7 (23.7) (*n* = 504)	n/a	<0.001
Mean time to cholangiography, min (SD)	45.2 (22.1) (*n* = 570)	40.8 (22.1) (*n* = 329)	1,105	0.004
Mean time to cholangiography^a^, min (SD)	42.3 (18.4) (*n* = 531)	37.9 (16.9) (*n* = 311)	n/a	<0.001
Intraoperative complications	16 (1.1)	3 (0.5)	n/a	0.25
Bile duct injury	6 (0.4)	3 (0.5)	n/a	0.69
Postoperative complications^1^	89 (6.1)	33 (6.0)	n/a	0.93
• Bleeding^b^	9 (0.6)	4 (0.7)	n/a	0.78
• Infection^c^	28 (1.9)	10 (1.8)	n/a	0.88
• Pancreatitis	26 (1.8)	10 (1.8)	n/a	0.96
• Bile leakage^d^	8 (0.5)	3 (0.5)	n/a	0.99
Readmission	78 (5.4)	42 (7.7)	n/a	0.054

ICG, indocyanine green; LC, laparoscopic cholecystectomy; SD, standard deviation.

^1^ Postoperative complications registered at 30 days follow-up.

^a^ Operations where operation time exceeded 120 min are excluded.

^b^ Defined as postoperative bleeding requiring intervention such as blood transfusion or surgery.

^c^ Defined as an infection requiring postoperative antibiotics, percutaneous drainage, or reoperation. Antibiotics started preoperatively or during surgery are not regarded as postoperative.

^d^ Defined as bile leakage requiring reoperation or ultrasound-guided drainage.

Intraoperative complications occurred in 19 cases; nine patients had a BDI (8 with <1/3 of the circumference and 1 with >1/3), 3 cases of intestinal injury, 4 cases of bleeding and 3 cases classified as “other”. There were no significant differences between groups regarding complications, but a non-significant difference was seen for readmission within 30 days (see Table 2).

### Conversion rate

Conversion rates were lower when ICG was used (1.2% vs. 3.3%, *p* = 0.001) and in elective surgeries (1.3% vs. 2.4%, *p* = 0.046). Patients undergoing converted procedures were significantly older (see Table 3). Neither gender nor BMI was significantly associated with risk for conversion.

**Table 3:** Conversions registered. Numbers in parentheses are corresponding percentages.

	Conversion	Not converted	*p*-value
ICG use:			0.001
- ICG used	17 (1.2)	1,438 (98.8)	
- ICG not used	18 (3.3)	531 (96.7)	
Gender, number (per cent)			0.087
- Female	17 (1.4)	1,238 (98.6)	
- Male	18 (2.4)	736 (97.6)	
Age, years (mean)	58.63	51.53	0.012
Surgery type			0.046
- Urgent	20 (2.4)	798 (97.6)	
- Elective	15 (1.3)	1,176 (98.7)	
BMI (mean)	29.02	29.26	0.81

In logistic regression, high age, urgent surgery, and non-use of ICG remained significant determinants of risk for conversion. The use of ICG decreased the risk for conversion with an OR of 0.31 (*p* < 0.001). Elective surgery was associated with a lower risk for conversion, OR 0.5 (*p* = 0.047). High age was associated with increased risk for conversion, with an OR of 1.03 (lower bound 1.01 and upper bound 1.06) for each year increase in age (*p* = 0.004).

### Operation time and time to intraoperative cholangiography

The mean operation time for all procedures was 70.7 (SD 37.7) min (see Table 2). Procedures performed without ICG had a mean operation time of 65.8 min compared to 72.6 min with ICG. The mean time to cholangiography was also significantly shorter in operations without ICG (40.8 and 45.2 min respectively). Time to cholangiography was missing in 1,105 patients. The distribution of missing data regarding time to cholangiography was 61 and 43 percent when ICG was used and in operations without ICG, respectively. The longer operation time and time to cholangiography when ICG was used persisted after exclusion of operations longer than 120 min.

Both operation time and time to cholangiography showed an increasing trend during the study period. The mean operation time 2015–2018 varied between 61 and 67 min, whereas it varied between 72 and 78 min 2019–2023. Mean time to IOC 2015–2018 ranged from 40 to 44 min whereas during 2019–2021 the mean time to IOC ranged between 49 and 53 min. In contrast to total operation time, the mean time until IOC fell over the last 2 years of the study, with mean times until IOC of 43 and 41 min in 2022 and 2023 respectively.

Factors identified as significant determinants of operation time were age, type of surgery (elective or acute), gender, and use of ICG. The use of ICG was negatively associated with operation time. In multivariable analysis with year of surgery included, all other factors remained significant apart from ICG use that was no longer associated with operation time. The results of multivariable linear regression analyses are presented in Table 4.

**Table 4:** Multivariable analysis with operation time as outcome.

Without year of surgery in the model	Unstandardized B	*p*-value	95% CI, lower bound	95% CI, upper bound
Constant	69.11	<0.001	62.82	75.4
ICG performed	6.08	<0.001	2.66	9.5
Age	0.27	<0.001	0.17	0.36
Elective operation	−22.24	<0.001	−25.34	−19.15
Gender (female)	−5.28	0.001	−8.49	−2.07
With year of surgery in the model				
Constant	67.87	<0.001	61.56	74.17
ICG performed	0.862	0.7	−3.53	5.26
Age (years)	0.26	<0.001	0.17	0.36
Elective operation	−21.03	<0.001	−24.19	−17.88
Gender (female)	−5.32	0.001	−8.51	−2.12
Year of operation				
- 2015–2018	Excluded	<0.001	3.54	11.58
- 2019–2023	7.56			

Multivariable analysis of determinants of Operation time.

CI, confidence interval, ICG, indocyanine green.

Regarding time to cholangiography, the same factors were associated as with operation time. Higher age was associated with longer time until cholangiography, while elective surgery and female gender was associated with shorter time. The use of ICG was negatively associated with time to cholangiography, and the association remained significant even in multivariable analysis including year of surgery. The covariables affecting time to cholangiography are presented in Table 5.

**Table 5:** Multivariable analysis with time to intraoperation cholangiography as outcome.

Without year of surgery in the model	Unstandardized B	*p*-value	95% CI, lower bound	95% CI, upper bound
Constant	44.89	<0.001	38.98	50.8
ICG performed	4.31	0.004	1.34	7.27
Age (years)	0.09	0.05	0	0.18
Elective operation	−9.14	<0.001	−12.19	−6.1
Gender (female)	−3.68	0.017	−6.7	−0.65
With year of surgery in the model				
Constant	44.96	<0.001	39.04	50.88
ICG performed	4.93	0.013	1.06	8.79
Age (years)	0.09	0.049	0.001	0.18
Elective operation	−9.26	<0.001	−12.35	−6.18
Gender (female)	−3.67	0.018	−6.69	−0.64
Year of operation				
- 2015–2018	Excluded	0.62	−4.69	2.81
- 2019–2023	−0.94			

Multivariable analysis of determinants of Time to Intraoperative Cholangiography.

CI, confidence interval; ICG, indocyanine green.

## Discussion

This study explored the influence on several outcome factors when using ICG fluorescence during LC performed with the FF method. ICG administration was found to be efficient and safe. Over a four-year period, over 90% of procedures were performed using ICG without increase in complication rates. The use of ICG fluorescence was associated with a lower risk for conversion to open surgery possibly due to better identification of the bile ducts. Findings in this study together with previous results suggest that the use of ICG fluorescence has the potential to improve safety in LC.

The risk for BDI should always be taken into consideration when performing LC. In our study of 2009 patients BDI occurred in nine patients, and no difference between the study groups could be seen. In clinical studies, very large study populations are needed to reach sufficient power to assess BDI as an outcome because of the rarity of BDI. Many studies have therefore focused on alternative outcomes, for example operation time.

In our study, use of ICG did not reduce operation time. On the contrary, in the univariable analysis there was a significantly shorter operation time when ICG was not used. The observed prolonged operations time is inconsistent with multiple previous studies (7, 34–39). Our results persisted even after the exclusion of prolonged LCs (>120 min), showing that surgeries with extreme operation times had no influence on the results (Table 2). In multivariate analysis including year of surgery, the association between ICG use and a prolonged operation time disappeared (Table 4). Time to IOC showed the same trend, but ICG remained an independent covariate for prolonged time to IOC. We have no explanation for the tendency for operation times to increase over the period of the study. However, the fact that ICG use was not a risk factor for prolonged operation time in multivariable analysis might indicate that it was not the use of ICG as such but other factors that prolonged the procedures over time.

Shorter operation times may reflect improved visualization, but whether this is associated with greater safety is debatable. In a recent study on 150,509 cholecystectomies comparing performances of male and female surgeons, shorter operation times were associated with higher complication rates (41). A longer operation time may reflect a more cautious surgical approach, and short operation time should not necessarily be considered a quality measure in LC. Furthermore, multiple confounding factors such as grade of inflammation, timing of surgery, educational surgery and additional surgical interventions such as an intraoperative ERCP must be taken into consideration when interpreting operation times.

Nevertheless, it may seem reasonable to consider operation time as a factor reflecting improved anatomical visualization and therefore operation safety. Van den Bos et al. (39) studied the time to reach “Critical View of Safety” (CVS), a variable more subjective than total operation time or time to IOC. Both operation time and time to achievement of CVS was approximately 4 min shorter when using ICG. Although the clinical relevance of saving 4 min is doubtful, other studies have shown larger reductions in operation time ranging from 20 to 30 min (32, 34–38).

However, we do not propose ICG as a substitute for IOC. Intraoperative cholangiography is not used routinely in many countries, but in Sweden it is considered a standard safety measure. The improved visualization with ICG is proposed as an adjunct for the prevention of bile duct injury. The aim of IOC is to evaluate the bile duct anatomy, detect any bile duct injuries and to confirm or exclude bile duct stones. The properties of the ICG fluorescence, with the fluorescence penetrating a few millimeters of tissue, rather exclude that it could be used for these indications, since the deeper bile duct structures cannot be visualized.

To the best of our knowledge, this is the first study evaluating the use of ICG during FF-LC. In contrast to conventional dissection, the bile ducts are approached after the gallbladder has been detached from the liver. As mentioned previously, FF has been associated with better outcomes in terms of postoperative convalescence, complications, and reduced cost when compared to traditional LC (17–20, 42). These results need to be confirmed in a prospective study. It would also be interesting to compare the use of ICG in FF and in traditional LC, preferably in the form of an RCT with sufficient power.

Consistent with previous studies (7, 37), we found a significantly lower conversion rate in the procedures with ICG (3.3% vs. 1.2%). The multivariable analysis revealed that ICG was the strongest determinant for conversion. This could reflect the better anatomical visualization that ICG provides, allowing the surgeon to proceed safely without the need to convert to open surgery. LCs are associated with less postoperative pain than open procedures, have shorter hospital stay, and fewer wound complications. The threefold higher conversion rate found in LCs without ICG in this study is highly relevant to the safety of patients being operated in the future.

There was a tendency to a reduced readmission rate in the group operated with ICG fluorescence (7.7 vs. 5.4%, *p* = 0.054). Late complications such as prolonged postoperative pain due to extensive tissue necrosis and minor bile leaks could contribute to this result and should be addressed in future studies. If confirmed, this would be another factor strongly supporting the advantages of ICG fluorescence in LC.

The most important strength of this study is the number of patients included. We have previously reported on how the implementation of FF as our standard for LC led to improved results compared to former methods (20). The relatively large population of patients undergoing FF LC using ICG with improved safety suggests that the present results are reliable. To our knowledge, this is one of the largest retrospective studies examining the impact of ICG fluorescence on outcome. A prospective study is being planned to further investigate some of these results.

The limitation of retrospectively collected data is evident in this study. Additional data are hard to acquire when searching for possible confounders, such as intraoperative ERCP. An ERCP procedure takes approximately 20–30 min which significantly increases operation time. Data regarding ERCP was not complete in our data and could therefore not be included in the multivariable analysis. Another variable with a rather large amount of missing data was time to intraoperative cholangiography, that was missing in 1,105 patients. Time to cholangiography is an outcome that we found especially relevant since it is a measurement that is well-defined since it can be found on the radiography images. Furthermore, concomitant surgeries such as an umbilical hernia repair might affect the overall operation time but not the time to cholangiography. Notwithstanding the missing data but we still found the data to be relevant. Furthermore, the missing data regarding cholangiography was 43% in the non-ICG group and 61% in the ICG group. We therefore considered the missing data to be acceptably distributed in the two groups.

By double-checking patient's notes regarding ICG administration we confirmed that ICG was used. However, time of ICG administration varied from 18 min to 25.2 h prior to surgery, and this could have influenced the overall results in the ICG group. The optimal timing of ICG administration has been studied with varying results. Furthermore, different imaging systems have been used, complicating the interpretation of these studies (43). However, many studies have shown that a longer time between injection and LC improves visualization (43–45). In these studies, doses equivalent to the doses used routinely at our hospital have been used.

Unfortunately, details on how much ICG was given were not available, and it is not known whether time and amount of ICG administered can impact results such as operation time, conversion rate, or complication rate. We also lacked information on whether monochromatic or continuous fluorescence imaging was used. ICG fluorescence can be used very briefly or for prolonged and repeated periods as is the surgeon's preference. Future studies with standard dosing, timing of ICG administration, clearer definition of ICG fluorescence technique, and quantification of its use are needed for better evaluation of ICG fluorescence in LC.

In conclusion, the present study shows that ICG fluorescence in FF-LC has the potential to reduce conversion rates. The implementation of ICG was efficient and safe. However, more controlled studies are needed to confirm the effectiveness of ICG and its role in reducing the risk for BDI.

## Data Availability

The datasets presented in this article are not readily available because the data contain information that could compromise the privacy of the research subjects. Data that support the findings of this study in pseudonymised form are available on reasonable request, from the corresponding author. Data are stored in the Swedish Gallstone Surgery and Endoscopic Retrograde Cholangiopancreatography Register, GallRiks [third party]. Requests to access the datasets should be directed to susanna.haverinen@ki.se.
